# Malnutrition Is Associated with Diabetic Retinopathy in Patients with Type 2 Diabetes

**DOI:** 10.1155/2023/1613727

**Published:** 2023-11-17

**Authors:** Wen Wei, Ruiyu Lin, Shihai Li, Zheyuan Chen, Qianqian Kang, Fenyan Lv, Wenying Zhong, Hangju Chen, Mei Tu

**Affiliations:** ^1^Department of Endocrinology, Fujian Longyan First Hospital, Longyan First Affiliated Hospital of Fujian Medical University, Longyan 364000, China; ^2^Department of Endocrinology, Zhujiang Hospital, Southern Medical University, Guangzhou 510280, China; ^3^Department of Endocrinology, Fujian Longyan First Hospital, Fujian Medical University, Fuzhou 350004, China; ^4^Department of Anesthesia, Fujian Longyan First Hospital, Longyan First Affiliated Hospital of Fujian Medical University, Longyan 364000, China; ^5^Department of Physical Examination, Fujian Longyan First Hospital, Longyan First Affiliated Hospital of Fujian Medical University, Longyan 364000, China

## Abstract

**Background:**

The relationship between malnutrition and diabetic retinopathy (DR) is still unclear. The purpose of this study is to investigate the relationship between malnutrition and DR in type 2 diabetic patients.

**Methods:**

A cross-sectional study was conducted on 612 patients with type 2 diabetes mellitus. Four malnutrition assessment tools: Global Leadership Initiative on Malnutrition (GLIM) criteria, controlling nutritional status (CONUT), nutritional risk index (NRI), and prognostic nutritional index (PNI), were applied to assess the nutritional status of the study population. The association between malnutrition and DR was examined using multivariable logistic regression and ordered logistic regression.

**Results:**

The proportion of malnutrition varied from 10.0% to 34.3% in total patients and from 16.3% to 45.1% in DR patients across the assessment tools. DR patients were more likely to be malnourished than patients without DR. The adjusted odds ratios (aOR) and 95% confidence interval (CI) for DR of malnutrition defined by different tools were 1.86 (1.01-3.14) for GLIM criteria, 1.67 (1.04-2.70) for NRI, and 2.24 (1.07-4.69) for PNI. The aOR and 95% CI for the severity of DR of malnutrition defined by different tools were 1.99 (1.12-3.51) for GLIM criteria, 1.65 (1.06-2.58) for NRI, and 2.51 (1.31-4.79) for PNI.

**Conclusions:**

Malnutrition was common in DR patients, and it was closely linked to the presence and severity of DR. Diabetic patients with DR should undergo nutritional assessment and early treatment of malnutrition to prevent the onset or progression of DR.

## 1. Introduction

Diabetic retinopathy (DR) is one of the major microvascular complications in patients with diabetes, affecting more than one-third of the diabetic population worldwide, and is the leading cause of preventable blindness in adults [1, 2]. In view of the health burden of DR complications on diabetic patients, identifying high-risk patients with DR and controlling risk factors for DR are crucial clinical tactics to keep patients with type 2 diabetes from developing visual impairment.

Previous evidence has shown that nutritional therapy can preserve the normal physiology, structure, and functions of the retina [3]. The Subjective Global Assessment (SGA) score, a subjective clinical nutrition assessment method, has been found to increase with the severity of DR [4]. Recent studies indicate that the prognostic nutritional index (PNI), which is obtained from serum albumin (ALB) and lymphocyte count, and the Geriatric Nutritional Risk Index (GNRI), which is calculated using height, weight, and ALB, are inversely and independently associated with the prevalence of DR [5, 6]. However, the relationship between malnutrition and the prevalence and severity of DR is still unclear.

Recently, consensus criteria for diagnosing malnutrition have been developed by the Global Leadership Initiative on Malnutrition (GLIM) and are broadly applicable. The GLIM approach is based on the evaluation of two etiologic (low food intake and the presence of disease with systemic inflammation) and three phenotypic (weight loss, low body mass index, and low skeletal muscle mass) criteria. The diagnosis can be confirmed by any combination of one etiologic and one phenotypic criterion being met [7]. The controlling nutritional status (CONUT) score and the nutritional risk index (NRI) are computed from low cost and easily obtained data such as ALB, total cholesterol (TC), lymphocyte count, height, and weight, which are relatively simple, practical, economical, and effective. Malnutrition, as screened by CONUT or NRI, is closely linked to a higher risk of cardiovascular events and death in patients with type 2 diabetes mellitus (T2DM) and acute coronary syndrome (ACS) [8, 9]. However, the three malnutrition assessment methods mentioned above have not been validated in patients with DR.

Therefore, we aim to use GLIM criteria, CONUT, NRI, and PNI nutritional assessment tools to screen for nutritional status in patients with DR and to evaluate the relationship between malnutrition and DR in patients with T2DM.

## 2. Methods

### 2.1. Study Population

**Figure 1:**
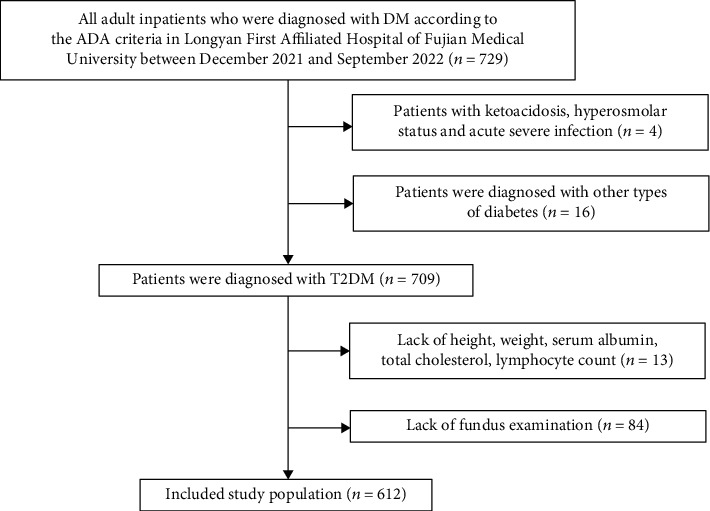
The flow of participants through the trial.

The present study was a cross-sectional study that included adult inpatients (≥18 years of age) diagnosed with T2DM according to American Diabetes Association (ADA) criteria at Longyan First Affiliated Hospital of Fujian Medical University, Fujian, China, between December 2021 and September 2022. Pregnant patients and patients with other types of diabetes were excluded. Patients with ketoacidosis, hyperosmolar status, acute severe infections, hemodialysis-dependent renal diseases, severe cardiac insufficiency, autoimmune diseases, and incomplete clinical parameters such as weight, height, serum albumin, total cholesterol, lymphocyte count, and fundus examination were also excluded. Eventually, 612 patients were enrolled (Figure 1). The Institutional Ethics Research Committee of Longyan First Affiliated Hospital of Fujian Medical University gave its approval to the study. All patients gave written informed consent to take part in the study.

### 2.2. Data Collection and Clinical Definition

Our trained staff collected demographic variables and health information, including age, sex, smoking status, alcohol consumption, and history of disease diagnosis, through standard questionnaires. Smoking status was defined as “current smoking” and “‘no current smoking.” Self-reported health conditions included the physician-diagnosed hypertension, stroke, and coronary artery disease (CAD).

Clinical features and biochemical examination data were obtained from the electronic medical record system. Height, weight, and blood pressure (BP) were assessed by the nurse on admission using a standardized form. Venous blood was collected early in the morning after fasting overnight [10]. Serum albumin, total cholesterol (TC), and lymphocyte count were measured as part of routine practice at the laboratory center in Longyan First Affiliated Hospital of Fujian Medical University.

Body mass index (BMI) was calculated using weight (kg) divided by the square of height (m^2^). Estimated glomerular filtration rate (eGFR) was calculated based on serum creatinine according to the Chronic Kidney Disease Epidemiology Collaboration (CKD-EPI) 2009 formula. Diabetic nephropathy (DN) was diagnosed based on the urinary albumin/creatinine ratio (ACR) ≥ 30 mg/mmol or eGFR < 60 mL/min/1.73 m^2^, as defined by the organization Kidney Disease Improving Global Outcomes (KDIGO) [11, 12]. Diabetic neuropathy was diagnosed according to the Chinese guidelines for the prevention and treatment of type 2 diabetes [13]. Atherosclerotic cardiovascular disease (ASCVD) was defined as a history of having any of CAD, stroke, peripheral arterial disease, or carotid arterial stenosis. Low education was defined as the patient's highest level of education being at or below a high school diploma.

### 2.3. Eye Examinations and Retinopathy Assessments

The diagnosis of DR was based on ophthalmologic examination, fundus fluorescein angiography (FFA) performed by specialized ophthalmologists, and the International Clinical Diabetic Retinopathy Disease Severity Scale [14]. Patients without DR were defined as those with no abnormalities on fundus photographs. Nonproliferative diabetic retinopathy (NPDR) includes intraretinal microaneurysms, hemorrhages, venous beading, and significant microvascular abnormalities. Proliferative diabetic retinopathy (PDR) involves neovascularization or vitreous/preretinal hemorrhages. Either NPDR or PDR was defined as diabetic retinopathy.

### 2.4. Malnutrition Screening Tools

We used GLIM criteria, CONUT score, NRI, and PNI to screen for malnutrition in our study.

The GLIM criteria are the consensus for the diagnosis of malnutrition [7]. A diagnosis of malnutrition could be confirmed by fulfilling at least one etiologic criterion and one phenotypic criterion. Etiologic criteria included a reduction in food intake or assimilation, as well as disease burden and inflammation. The assessment of “reduced food intake or assimilation” depends on self-reported information about actual intake compared to usual intake, and the presence of gastrointestinal symptoms or the presence of clinical diagnoses that adversely affects food assimilation. The criterion “presence of inflammation or disease severity” was established on the basis of ALB (<35 g/L) or neutrophil-to-lymphocyte ratio (NLR) (≥3 or <0.7) [15]. Phenotypic criteria applied in this study included low BMI and reduced muscle mass. A BMI of <18.5 kg/m^2^ in patients < 70 years or <20.0 kg/m^2^ in patients ≥ 70 years was considered a low BMI in the Asian populations. Reduced muscle mass was reflected by appendicular skeletal muscle mass index (ASMMI) of <7.0 kg/m^2^ for males or <5.4 kg/m^2^ for females [16]. Fat mass and muscle mass were measured using dual-energy X-ray absorptiometry (DXA). The patient's muscle mass was evaluated according to their ASMMI, which was expressed as the appendicular skeletal muscle mass (ASMM) divided by the square of height (m^2^).

The CONUT score was developed by Ulibarri et al. in 2005 as a screening tool to assess the nutritional status of hospitalized patients [17]. It automatically assesses the nutritional status through serum albumin, total cholesterol, and lymphocyte count. A score of 0 to 1 indicates normal nutrition status; scores of 2 to 4, 5 to 8, and 9 to 12 indicate mild, moderate, and severe malnutrition, respectively.

The NRI was described by Buzby et al. to screen for malnutrition in various medical and surgical patient populations [18]. The NRI was calculated using the formula 1.519^∗^ serum albumin (g/L) + 41.7^∗^ (current body weight [kg]/usual body weight [kg]). In line with previous research and utilizing the Lorenz formulas, usual body weight was replaced by ideal body weight—that is, height (cm)–100 − ([height (cm) − 150]/4) for men and height (cm)–100 − ([height (cm) − 150]/2.5) for women—and when the current body weight exceeds the ideal body weight, the current body weight/ideal body weight = 1 [19, 20]. The NRI ≥ 100 reflects normal nutrition status; 97.5 ≤ NRI < 100, 83.5 ≤ NRI < 97.5, and NRI < 83.5 reflect mild, moderate, and severe malnutrition, respectively.

The PNI was calculated using the following formula: serum albumin (g/L) + 5^∗^ lymphocyte count (10^9^/L) [21, 22]. A score of ≥43.0 is regarded as normal; scores of 38.0 to 42.9 and <38.0 reflect moderate and severe malnutrition, respectively [23]. There is no mild classification of the PNI.

### 2.5. Statistical Analysis

Data were presented as mean ± standard deviation or median (interquartile range) depending on the distribution of the continuous variable, and categorical variables were presented as frequencies or percentages. For comparisons between groups, the *χ*^2^ test or Fisher's exact test was used for categorical variables, and one-way analysis of variance (ANOVA) was used for continuous variables, as appropriate.

Restricted cubic splines were used to detect the association between the three malnutrition screening tools and DR. The association between malnutrition and DR was assessed by univariable and multivariable logistic regressions. The ordered logistic regression was applied to analyze the relationship between malnutrition and the severity of DR. Variables that were entered into the model were carefully selected on the basis of variables associated with known poor prognosis or variables with *p* value < 0.05 in the baseline or univariable regression analysis.

Statistical analyses were performed using R, version 4.0.3 software (R Foundation for Statistical Computing, Vienna, Austria) and SPSS version 26.0 (IBM Corp., Armonk, NY). Two-sided *p* values < 0.05 were considered statistically significant.

## 3. Results

### 3.1. Clinical Characteristics

Of the 612 patients included in the study, the majority were male (62.6%), and the average age was 57.6 years. In total, the current smokers were 169 (27.7%), and patients with low education were 510 (83.3%). The median duration of diabetes was 6.0 years, and the glycosylated hemoglobin (HbA1c) was 9.9% ± 2.4. Almost half of the patients had hypertension (44.6%; *n* = 273), 15.4% (*n* = 94) had DN, 43.5% (*n* = 266) had diabetic neuropathy, and 10.5% (*n* = 64) had ASCVD. 110 (18.5%) patients were treated with insulin and 390 (65.3%) with an oral antidiabetic drug (OAD) (Table 1). The prevalence of NPDR was 17.6%, and PDR was 12.4%. Patients were divided into three groups according to their DR status. Compared with patients without DR, patients with NPDR and PDR were older; had lower BMI, lower serum albumin levels, and lower eGFR; were accompanied with higher systolic blood pressure (SBP); and had longer duration of diabetes. They also had higher prevalence of hypertension, stroke, DN, diabetic neuropathy, and ASCVD than patients without DR. The use of OAD, insulin, and ACEI/ARB was higher in patients with NPDR and PDR than in patients without DR (Table 2 and Supplementary Table 1).

Regardless of which nutritional assessment method was used, patients with malnutrition tended to be older, had lower BMI, and had higher prevalence of DN and anemia than patients with nonmalnutrition. Additionally, patients with malnutrition had lower eGFR, albumin, and lymphocyte levels, accompanied with higher hs-CRP and neutrophil levels (Supplementary Tables 2–5).

### 3.2. Prevalence of Malnutrition

The percentage of patients with malnutrition varied, with a total of 18.2% malnourished according to the GLIM criteria, 10.0% according to the PNI, 29.9% according to the CONUT score, and 34.3% according to the NRI. Using the CONUT and NRI scores, 157 (25.7%) and 77 (12.6%) patients had mild malnutrition, respectively. Based on CONUT, NRI, PNI, and GLIM criteria, 26 (4.2%), 133 (21.7%), 61 (10.0%), and 106 (18.2%) patients had moderate to severe malnutrition, respectively (Table 1).

**Figure 2:**
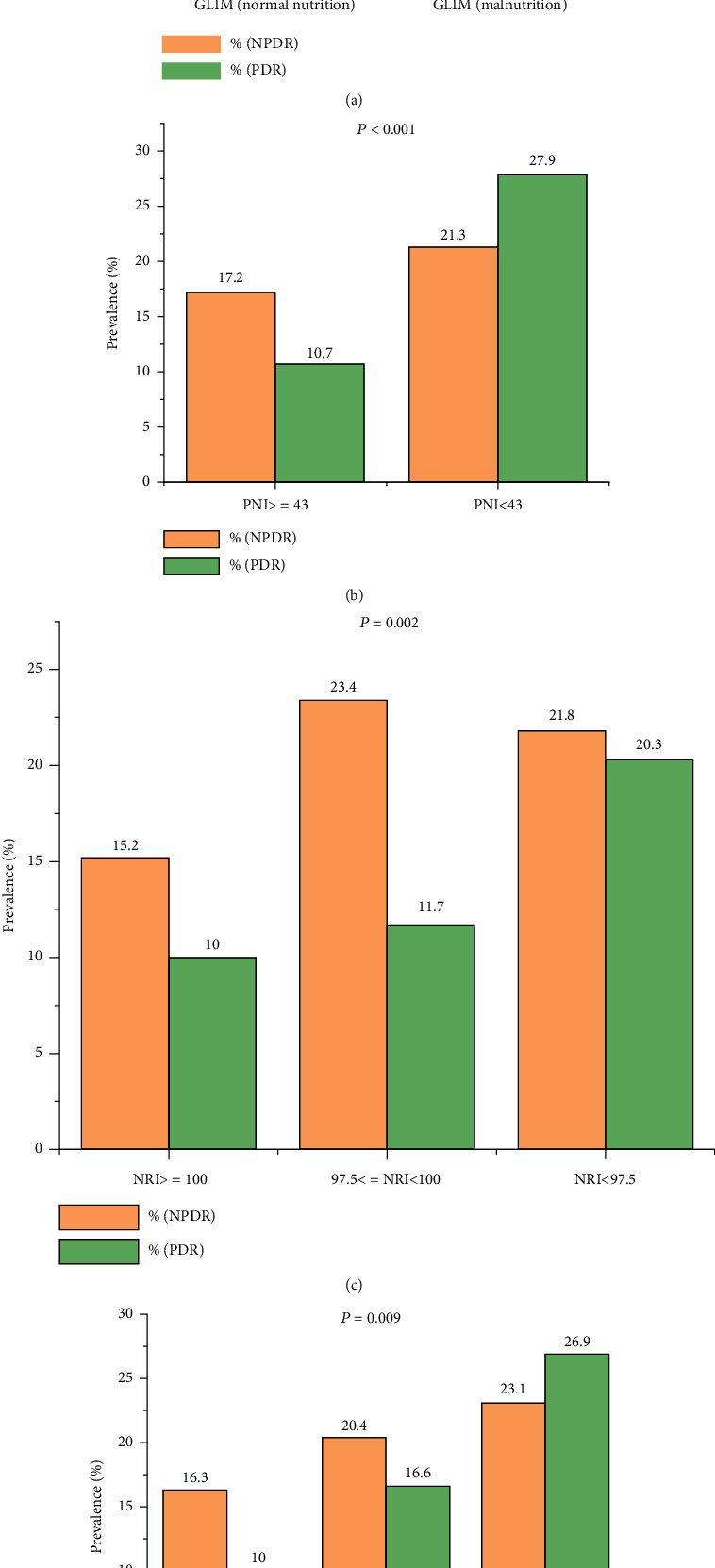
Prevalence of NPDR and PDR in different nutritional states. (a) Prevalence of NPDR and PDR in different nutritional states measured by GLIM criteria. (b) Prevalence of NPDR and PDR in different nutritional states measured by PNI. (c) Prevalence of NPDR and PDR in different nutritional states measured by NRI. (d) Prevalence of NPDR and PDR in different nutritional states measured by CONUT.

In patients with DR, the prevalence of malnutrition was 24.0% with the GLIM criteria, 16.3% with the PNI, 38.6% with the CONUT score, and 45.1% with the NRI. Irrespective of the malnutrition tool used, patients with DR showed higher incidence of malnutrition than patients without DR (Table 2). Conversely, the incidence of DR in malnourished patients was also higher than that in patients with normal nutritional status (Figure 2).

### 3.3. Association between Malnutrition and Diabetic Retinopathy

**Figure 3:**
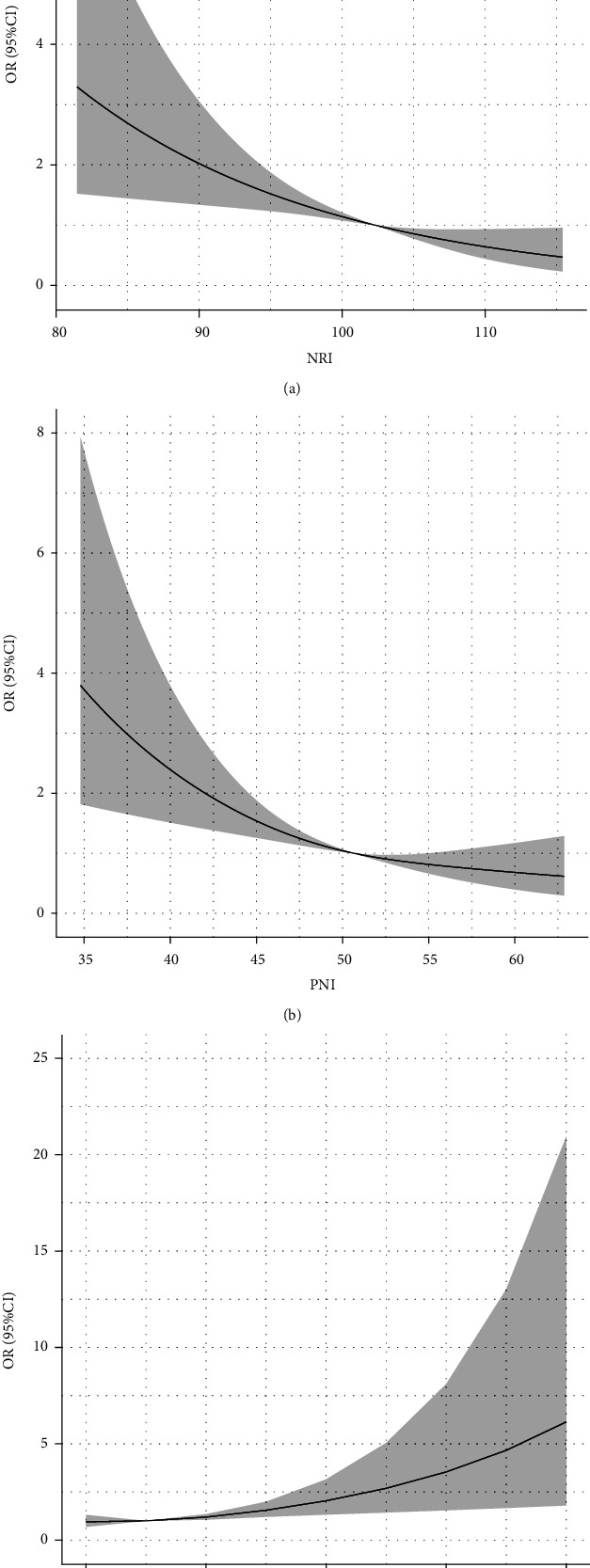
Restricted spline curve of the malnutrition screening tool odds ratio of DR. (a) The restricted spline curve of NRI odds ratio of DR. (b) The restricted spline curve of PNI odds ratio of DR. (c) The restricted spline curve of CONUT odds ratio of DR.

There was a linear correlation between malnutrition screening tools and DR in our study population. We found that the higher the NRI or PNI, the lower the incidence of DR. Conversely, the higher the CONUT score, the higher the incidence of DR (nonlinear: NRI, *p* = 0.993; PNI, *p* = 0.327; CONUT score, *p* = 0.349) (Figure 3).

Multivariable logistic regression showed an independent association between higher NRI and PNI levels and lower incidence of DR in T2DM patients (NRI—adjusted odds ratio [aOR]: 0.95, 95% confidence interval [CI]: 0.92-0.99; PNI—aOR: 0.96, 95% CI: 0.92-1.00). Patients with malnutrition, as measured by GLIM criteria, NRI, or PNI, had a higher risk of DR than those with normal nutritional status (GLIM criteria—aOR: 1.86, 95% CI: 1.01-3.14; NRI—aOR: 1.67, 95% CI: 1.04-2.70; PNI—aOR: 2.24, 95% CI: 1.07-4.69). Age, gender, BMI, smoking, duration of diabetes, hypertension, low-density lipoprotein cholesterol (LDL-C), hypersensitive C-reactive protein (hs-CRP), anemia, diabetic neuropathy, DN, ASCVD, low education, OAD, insulin, and ACEI/ARB were included as covariates in the multivariable analysis. Both the CONUT score and malnutrition assessed by the CONUT score were strongly correlated with DR when not adjusting for covariates. However, the correlation gradually disappeared after adjusting for covariates (Table 3 and Supplementary Table 6).

Moreover, the ordered logistic regression analysis showed that malnutrition, as measured by GLIM criteria, NRI, or PNI, was associated with the severity of DR (GLIM criteria—ordinal OR: 1.99, 95% CI: 1.12-3.51; NRI—ordinal OR: 1.65, 95% CI: 1.06-2.58; PNI—ordinal OR: 2.51, 95% CI: 1.31-4.79). The models passed the parallelism test (*p* > 0.05).

## 4. Discussion

To the best of our knowledge, this present study was the first study to investigate the association between malnutrition, as assessed by various screening tools, and DR in patients with T2DM. Our study found that patients with DR tended to be more malnourished than those without DR, and patients with malnutrition had higher incidence of DR than those with normal nutritional status. Malnutrition was independently associated with the presence and severity of DR.

There are only a few studies of malnutrition prevalence on patients with DR. Our study revealed that the prevalence of malnutrition was higher in patients with DR than in those without DR. The percentages were 38.6% with the CONUT score, 45.1% with the NRI, 16.3% with the PNI, and 24.0% with the GLIM criteria. Although the proportion of malnutrition assessed by different assessment tools varied widely, the prevalence of malnutrition in DR remained high overall. Diabetic patients with malnutrition tend to have infections, diabetic foot ulcers (DFU), diabetic kidney disease (DKD), and coronary artery disease (CAD) and have a poor prognosis [24–27]. It is well known that DR is one of the common microvascular complications of diabetes. Our study also revealed that malnutrition was associated with higher incidence of DR.

In previous studies, Cho et al. included T2DM patients from the diabetes clinic and found that patients who were in a lower tertile of the GNRI tended to have a higher prevalence of DR compared to those with higher GNRIs [6]. Yang et al. enrolled hospitalized T2DM patients and found a significant association between higher PNI levels and lower prevalence of DR [5]. Similarly, Kurtul et al. reported that a lower PNI value was strongly associated with the occurrence of DR. The sensitivity of PNI for predicting DR was 74%, and the specificity was 64% [28]. The GLIM criteria are a new method for evaluating malnutrition. A recent study in Saudi Arabia found that GLIM criteria and SGA were very consistent in diagnosing malnutrition in T2DM patients (GLIM: 15.8%; SGA: 17.8%) [29]. According to the GLIM criteria, malnutrition was found in 18.2% of patients in our study, which was similar to the study conducted in Saudi Arabia. Different from previous studies, we included PNI, NRI, and CONUT as continuous variables in the analysis. Additionally, we assessed malnutrition status using various malnutrition screening tools. Moreover, we simultaneously utilized four malnutrition assessment tools. The results showed that higher NRI and PNI levels were independently linked to lower prevalence of DR, and malnutrition, as measured by GLIM criteria, NRI, or PNI, was independently linked to the presence of DR after adjusting for covariates such as age, gender, BMI, smoking, low education, duration of diabetes, hypertension, LDL-C, hs-CRP, anemia, DPN, DN, ASCVD, OAD, insulin, and ACEI/ARB. In addition, we also analyzed the correlation between malnutrition and the severity of DR, and the study demonstrated that malnutrition and the severity of DR were independently correlated after adjusting for the same covariates described above.

The performance of the CONUT score was not as good as other tools. The majority of individuals identified as malnourished by the CONUT score were classified as mildly malnourished. This could be attributed to statins reducing total cholesterol levels, which may lead to an overestimation of the likelihood of mild malnutrition. Moreover, the univariable analysis revealed no correlation between total cholesterol levels and DR. Therefore, our findings suggested that the CONUT score may not be an ideal tool to assess nutritional status in patients with DR. According to the NRI score, mild malnutrition had a neutral impact on DR, and only moderate-severe malnutrition was strongly associated with an increased risk of DR. The absence of mild categories is a limitation of the clinical application of the PNI and GLIM standards, and there is no consensus on the optimal cutoff value of PNI or the corresponding criterion “presence of inflammation or disease severity” in the GLIM criteria. Larger prospective studies are needed to confirm the predictive value of different nutritional assessment tools in DR.

Previous studies showed that patients with DR were likely to be elderly, be men, and have higher BMI [30]. Our study showed that patients with DR or malnutrition were elderly, which is consistent with previous research. In terms of gender, there was no statistical difference in the proportion of men with or without DR in our study. The proportion of malnourished patients assessed by GLIM, PNI, and CONUT was higher in males. In the current study, gender differences in DR and malnutrition are inconclusive [31–33]. In our study, patients with DR had a lower BMI, which was contrary to the Yin et al.'s study [30]. However, the current meta-analysis showed that there was no difference in the prevalence of DR between obese and nonobese diabetic patients [31]. In our study, malnourished patients had lower BMI, which is consistent with most previous studies. According to GLIM diagnostic criteria, sarcopenic obesity was also present in malnutrition. Therefore, gender differences in DR and malnutrition, as well as the relationship between obesity and DR, need further research. Previous studies showed that diabetics with low education were more likely to develop DR, and low education also increased the risk of malnutrition [32, 34]. However, in our study, educational attainment did not differ among diabetic patients with or without DR, and the prevalence of low education was only higher in the malnutrition assessed by the NRI. This may be related to the low educational attainment of the majority of patients in our study, and further research is needed to verify this issue. Taking into account the influence of these confounders, we adjusted for age, gender, BMI, and low education in regression analysis, and the findings still showed a significant association between malnutrition and DR. Previous studies found that Africans had the highest prevalence of DR, Asians had the lowest prevalence of DR, and the gap in the incidence of DR between whites and blacks was narrowing [35, 36]. The prevalence of malnutrition among different ethnic groups was not known. Further studies on the relationship between malnutrition and DR in different regions and ethnic groups are needed to confirm our findings.

The traditional view is that hyperglycemia, hypertension, dyslipidemia, inflammation, and anemia are the related factors of DR [37, 38]. There is growing evidence that malnutrition is a potential risk factor for DR. Nutritional strategies can reduce the risk of developing DR, proving quite beneficial in cases of DR that are resistant to conventional medical treatments [3]. Considering the high prevalence of malnutrition in patients with type 2 diabetes and DR, and the fact that malnutrition is a modifiable risk factor, we recommend conducting nutritional assessments for all diabetic patients, especially those with DR. This will help identify individuals who are malnourished and allow for early treatment of malnutrition, thereby preventing the occurrence or progression of DR.

Our research has several limitations. First, the cross-sectional design of this study makes it difficult to determine the causal relationship between malnutrition and DR. Future prospective cohort studies are needed to explore the causal relationship between malnutrition and DR and to evaluate how different nutritional assessment tools predict outcomes of DR. Second, due to insufficient data on weight loss, we had to simplify the GLIM criteria, which may underestimate the prevalence of malnutrition assessed by the GLIM criteria. Third, the study was limited to inpatients with type 2 diabetes, and whether the findings can be applied to outpatients or community-dwelling ones with type 2 diabetes remains unknown. Furthermore, this study was conducted at a single center in China, which may limit the generalizability of the findings to other regions with different economic and cultural levels. These need to be studied in the future.

## 5. Conclusion

The prevalence of malnutrition varied with different assessment tools, but malnutrition was common in T2DM patients with DR. Worsening nutritional status was independently associated with the presence and severity of DR. Longitudinal studies involving diverse racial groups should be conducted to determine the causal relationship between nutritional status and DR, as well as to assess the effectiveness of different nutritional assessment tools in predicting DR outcomes.

## Figures and Tables

**Table 1:** Baseline characteristics of all participants.

	All participants (*n* = 612)
Demographic characteristics	
Age (years)	57.6 ± 11.8
Age ≥ 60, *n* (%)	256 (41.8)
Male, *n* (%)	383 (62.6)
BMI (kg/m^2^)	24.3 ± 3.5
BMI ≥ 24 kg/m^2^, *n* (%)	294 (48.0)
Smoking, *n* (%)	169 (27.7)
Low education, *n* (%)	510 (83.3)

Medical history and clinical condition	
Hypertension, *n* (%)	273 (44.6)
SBP (mmHg)	133.0 ± 18.6
DBP (mmHg)	82.7 ± 11.7
Duration of diabetes (years)	6.0 (1, 10)
DN, *n* (%)	94 (15.4)
DPN, *n* (%)	266 (43.5)
CAD, *n* (%)	45 (7.4)
Stroke, *n* (%)	23 (3.8)
ASCVD, *n* (%)	64 (10.5)
Anemia, *n* (%)	111 (18.1)

Laboratory examination	
FBG (mmol/L)	8.69 ± 3.21
2hPBG (mmol/L)	11.48 ± 4.24
HbA1C (%)	9.89 ± 2.42
TG (mmol/L)	1.60 (1.08, 2.63)
TC (mmol/L)	5.18 ± 1.50
LDL-C (mmol/L)	3.23 ± 1.00
HDL-C (mmol/L)	1.09 ± 0.38
eGFR (mL/min/1.73 m^2^)	92.02 ± 22.48
hs-CRP (mg/L)	1.60 (0.70, 3.50)
Albumin (g/L)	40.32 ± 4.40
Hb (g/L)	139.00 ± 18.05
Lymphocyte (10^9^/L)	2.03 ± 0.67
Neutrophil (10^9^/L)	4.29 ± 1.58

Medication	
OAD, *n* (%)	390 (65.3)
Insulin, *n* (%)	110 (18.5)
ACEI/ARB, *n* (%)	115 (20.4)

Malnutrition	
GLIM, *n* (%)	106 (18.2)
PNI, continuous	50.48 ± 5.97
PNI, categorical, *n* (%)	61 (10.0)
NRI, continuous	102.02 ± 7.21
NRI, categorical, *n* (%)	
Mild	77 (12.6)
Moderate-severe	133 (21.7)
CONUT, continuous	1(0, 2)
CONUT, categorical, *n* (%)	
Mild	157 (25.7)
Moderate-severe	26 (4.2)

Abbreviations: BMI: body mass index; SBP: systolic blood pressure; DBP: diastolic blood pressure; DN: diabetic nephropathy; DPN: diabetic peripheral neuropathy; CAD: coronary artery disease; ASCVD: atherosclerotic cardiovascular disease; FBG: fasting blood glucose; 2hPBG: 2-hour postprandial blood glucose; HbA1c: glycosylated hemoglobin; TG: triglyceride; TC: total cholesterol; LDL-C: low-density lipoprotein cholesterol; HDL-C: high-density lipoprotein cholesterol; eGFR: estimated glomerular filtration rate; hs-CRP: hypersensitive C-reactive protein; OAD: oral antidiabetic drug; ACEI/ARB: angiotensin-converting enzyme inhibitor/angiotensin receptor blocker; GLIM: Global Leadership Initiative on Malnutrition; PNI: prognostic nutritional index; NRI: nutritional risk index; CONUT: controlling nutritional status.

**Table 2:** Baseline characteristics of patients with different retinopathy statuses.

	Non-DR (*n* = 428)	NPDR (*n* = 108)	PDR (*n* = 76)	*p* value
Demographic characteristics				
Age (years)	56.9 ± 12.2	58.9 ± 10.6	59.5 ± 11.0	0.103
Age ≥ 60, *n* (%)	173 (40.4)	51 (47.2)	32 (42.1)	0.440
Male, *n* (%)	277 (64.7)	63 (58.3)	43 (56.6)	0.242
BMI (kg/m^2^)	24.5 ± 3.6	23.7 ± 3.1	23.9 ± 3.0	0.067
BMI ≥ 24 kg/m^2^, *n* (%)	213 (49.8)	49 (45.4)	32 (42.1)	0.388
Smoking, *n* (%)	131 (30.7)	26 (24.1)	12 (15.8)	0.018
Low education, *n* (%)	353 (82.5)	93 (86.1)	64 (84.2)	0.648

Medical history and clinical condition				
Hypertension, *n* (%)	179 (41.8)	58 (53.7)	36 (47.4)	0.074
SBP (mmHg)	131.3 ± 17.9	135.6 ± 19.8	138.6 ± 19.6	0.002
DBP (mmHg)	82.6 ± 11.7	83.4 ± 11.3	82.2 ± 12.0	0.756
Duration of diabetes (years)	4 (0, 10)	10 (6, 17)	10 (7, 18)	<0.001
DN, *n* (%)	42 (9.8)	25 (23.1)	27 (35.5)	<0.001
DPN, *n* (%)	148 (34.6)	70 (64.8)	48 (63.2)	<0.001
CAD, *n* (%)	30 (7.0)	9 (8.3)	6 (7.9)	0.878
Stroke, *n* (%)	7 (1.6)	10 (9.3)	6 (7.9)	<0.001
ASCVD, *n* (%)	35 (8.2)	18 (16.7)	11 (14.5)	0.017
Anemia, *n* (%)	58 (13.6)	28 (25.9)	25 (32.9)	<0.001

Laboratory examination				
FBG (mmol/L)	8.75 ± 3.20	8.35 ± 3.02	8.80 ± 3.52	0.487
2hPBG (mmol/L)	11.54 ± 4.30	11.55 ± 4.09	11.00 ± 4.12	0.588
HbA1C (%)	9.92 ± 2.45	10.08 ± 2.40	9.41 ± 2.21	0.165
TG (mmol/L)	1.63 (1.07, 2.73)	1.54 (1.15, 2.30)	1.53 (1.03, 2.47)	0.748
TC (mmol/L)	5.22 ± 1.55	4.93 ± 1.41	5.27 ± 1.35	0.165
LDL-C (mmol/L)	3.28 ± 1.01	3.02 ± 0.95	3.25 ± 0.97	0.058
HDL-C (mmol/L)	1.09 ± 0.38	1.09 ± 0.25	1.15 ± 0.49	0.391
eGFR (mL/min/1.73 m^2^)	94.95 ± 20.33	89.83 ± 23.11	78.66 ± 27.65	<0.001
hs-CRP (mg/L)	1.70 (0.70, 3.40)	1.30 (0.50, 3.50)	1.35 (0.60, 3.55)	0.595
Albumin (g/L)	40.89 ± 4.02	39.52 ± 4.18	38.27 ± 5.79	<0.001
Hb (g/L)	141.42 ± 16.54	136.49 ± 17.51	128.95 ± 22.63	<0.001
Lymphocyte (10^9^/L)	2.06 ± 0.67	2.01 ± 0.71	1.93 ± 0.61	0.289
Neutrophil (10^9^/L)	4.25 ± 1.56	4.42 ± 1.73	4.34 ± 1.47	0.578

Medication				
OAD, *n* (%)	258 (61.6)	77 (74.0)	55 (74.3)	0.013
Insulin, *n* (%)	47 (11.2)	35 (33.7)	28 (37.8)	<0.001
ACEI/ARB, *n* (%)	73 (18.4)	27 (28.4)	15 (20.3)	0.095

Malnutrition				
GLIM, *n* (%)	65 (15.8)	18 (17.8)	23 (32.9)	0.003
PNI, continuous	51.17 ± 5.56	49.56 ± 6.15	47.92 ± 7.10	<0.001
PNI, categorical, *n* (%)	31 (7.2)	13 (12.0)	17 (22.4)	<0.001
NRI, continuous	102.92 ± 6.73	100.51 ± 7.00	99.07 ± 8.90	<0.001
NRI, categorical, *n* (%)				0.002
Mild	50 (11.7)	18 (16.7)	9 (11.8)	
Moderate-severe	77 (18.0)	29 (26.9)	27 (35.5)	
CONUT, continuous	1 (0, 2)	1 (0, 2)	1 (0, 3)	0.015
CONUT, categorical, *n* (%)				0.009
Mild	99 (23.1)	32 (29.6)	26 (34.2)	
Moderate-severe	13 (3.0)	6 (5.6)	7 (9.2)	

Abbreviations: DR: diabetic retinopathy; NPDR: nonproliferative diabetic retinopathy; PDR: proliferative diabetic retinopathy; BMI: body mass index; SBP: systolic blood pressure; DBP: diastolic blood pressure; DN: diabetic nephropathy; DPN: diabetic peripheral neuropathy; CAD: coronary artery disease; ASCVD: atherosclerotic cardiovascular disease; FBG: fasting blood glucose; 2hPBG: 2-hour postprandial blood glucose; HbA1c: glycosylated hemoglobin; TG: triglyceride; TC: total cholesterol; LDL-C: low-density lipoprotein cholesterol; HDL-C: high-density lipoprotein cholesterol; eGFR: estimated glomerular filtration rate; hs-CRP: hypersensitive C-reactive protein; OAD: oral antidiabetic drug; ACEI/ARB: angiotensin-converting enzyme inhibitor/angiotensin receptor blocker; GLIM: Global Leadership Initiative on Malnutrition; PNI: prognostic nutritional index; NRI: nutritional risk index; CONUT: controlling nutritional status.

**Table 3:** Associations of malnutrition assessed by four assessment tools with DR.

	Univariable	*p* value	Multivariable	*p* value
	OR (95% CI)		aOR (95% CI)	
GLIM (normal nutrition as reference)	1.68 (1.08-2.61)	0.020	1.86 (1.01-3.14)	0.046
PNI, continuous	0.94 (0.91-0.97)	<0.001	0.96 (0.92-1.00)	0.033
PNI, categorical (normal nutrition as reference)	2.50 (1.46-4.27)	0.001	2.24 (1.07-4.69)	0.032
NRI, continuous	0.94 (0.92-0.97)	<0.001	0.95 (0.92-0.99)	0.007
NRI, categorical (normal nutrition as reference)				
Malnutrition	1.95 (1.36-2.78)	<0.001	1.67 (1.04-2.70)	0.034
Mild	1.61 (0.95-2.69)	0.073	1.50 (0.79-2.79)	0.207
Moderate-severe	2.17 (1.43-3.27)	<0.001	1.83 (1.03-3.23)	0.038
CONUT, continuous	1.22 (1.09-1.37)	0.001	1.14 (0.95-1.36)	0.149
CONUT, categorical (normal nutrition as reference)				
Malnutrition	1.77 (1.23-2.56)	0.002	1.39 (0.81-2.36)	0.228
Mild	1.64 (1.11-2.41)	0.013	1.31 (0.75-2.25)	0.339
Moderate-severe	2.80 (1.25-6.27)	0.012	2.35 (0.77-7.27)	0.134

Adjusted for age, gender, BMI, smoking, low education, duration of diabetes, hypertension, LDL-C, hs-CRP, anemia, DPN, DN, ASCVD, OAD, insulin, and ACEI/ARB. Abbreviations: DR: diabetic retinopathy; GLIM: Global Leadership Initiative on Malnutrition; PNI: prognostic nutritional index; NRI: nutritional risk index; CONUT: controlling nutritional status; BMI: body mass index; LDL-C: low-density lipoprotein cholesterol; hs-CRP: hypersensitive C-reactive protein; DPN: diabetic peripheral neuropathy; DN: diabetic nephropathy; ASCVD: atherosclerotic cardiovascular disease; OAD: oral antidiabetic drug; ACEI/ARB: angiotensin-converting enzyme inhibitor/angiotensin receptor blocker.

## Data Availability

The data used to support the findings of this study have not been made available because of patient privacy.

## Supplementary Materials

Supplementary Materials Baseline characteristics of patients with and without DR, baseline characteristics of malnourished and nonmalnourished patients, and univariable logistic regression analysis for variables and DR were included in the supplementary materials. Supplementary Table 1: baseline characteristics of patients with and without DR. Supplementary Table 2: baseline characteristics of malnourished and nonmalnourished patients assessed by GLIM. Supplementary Table 3: baseline characteristics of malnourished and nonmalnourished patients assessed by PNI. Supplementary Table 4: baseline characteristics of malnourished and nonmalnourished patients assessed by NRI. Supplementary Table 5: baseline characteristics of malnourished and nonmalnourished patients assessed by CONUT. Supplementary Table 6: univariable logistic regression analysis for variables and DR.
