# Supplementary material for: Malnutrition Is Associated with Diabetic Retinopathy in Patients with Type 2 Diabetes
Source: J Diabetes Res. 2023 Nov 17;2023:1613727. doi: 10.1155/2023/1613727 (PMC10673668; doi:10.1155/2023/1613727)
Supplement: Supplementary Materials — Baseline characteristics of patients with and without DR, baseline characteristics of malnourished and nonmalnourished patients, and univariable logistic regression analysis for variables and DR were included in the supplementary materials. Supplementary Table 1: baseline characteristics of patients with and without DR. Supplementary Table 2: baseline characteristics of malnourished and nonmalnourished patients assessed by GLIM. Supplementary Table 3: baseline characteristics of malnourished and nonmalnourished patients assessed by PNI. Supplementary Table 4: baseline characteristics of malnourished and nonmalnourished patients assessed by NRI. Supplementary Table 5: baseline characteristics of malnourished and nonmalnourished patients assessed by CONUT. Supplementary Table 6: univariable logistic regression analysis for variables and DR. [file 1613727.f1.docx]

**Supplementary Table 1. Baseline characteristics of patients with and without DR.**

|  | Non-DR  （n=428） | DR  （n=184） | p-value |
| --- | --- | --- | --- |
| **Demographic characteristics** | | | |
| Age (years) | 56.9 ± 12.2 | 59.1 ± 10.7 | 0. 036 |
| Age≥60, n (%) | 173 (40.4) | 83 (45.1) | 0.323 |
| Male, n (%) | 277 (64.7) | 106 (57.6) | 0.115 |
| BMI (kg/m^2^) | 24.5 ± 3.6 | 23.8 ± 3.1 | 0.023 |
| BMI ≥ 24 kg/m^2^, n (%) | 213 (49.8) | 81 (44.0) | 0.224 |
| Smoking, n (%) | 131 (30.7) | 38 (20.7) | 0.015 |
| Low Education, n (%) | 353 (82.5) | 157 (85.3) | 0.454 |
| **Medical history and Clinical condition** | | | |
| Hypertension, n (%) | 179 (41.8) | 94 (51.1) | 0.043 |
| SBP (mmHg) | 131.3 ± 17.9 | 136.9 ± 19.7 | 0.001 |
| DBP (mmHg) | 82.6 ± 11.7 | 82.9 ± 11.6 | 0.787 |
| Duration of diabetes (years) | 4(0, 10) | 10(7, 18) | <0.001 |
| DN, n (%) | 42 (9.8) | 52 (28.3) | <0.001 |
| DPN, n (%) | 148 (34.6) | 118 (64.1) | <0.001 |
| CAD, n (%) | 30 (7.0) | 15 (8.2) | 0.743 |
| Stroke, n (%) | 7 (1.6) | 16 (8.7) | <0.001 |
| ASCVD, n (%) | 35 (8.2) | 29 (15.8) | 0.008 |
| Anemia, n (%) | 58 (13.6) | 53 (28.8) | <0.001 |
| **Laboratory examination** | | | |
| FBG(mmol/L) | 8.75 ± 3.20 | 8.54 ± 3.23 | 0.449 |
| 2hPBG(mmol/L) | 11.54 ± 4.30 | 11.33 ± 4.10 | 0.568 |
| HbA1C(%) | 9.92 ± 2.45 | 9.81 ± 2.34 | 0.589 |
| TG(mmol/L) | 1.63(1.07, 2.73) | 1.54(1.10, 2.41) | 0.464 |
| TC(mmol/L) | 5.22 ± 1.55 | 5.07 ± 1.39 | 0.242 |
| LDL-C(mmol/L) | 3.28 ± 1.01 | 3.12 ± 0.97 | 0.065 |
| HDL-C(mmol/L) | 1.09 ± 0.38 | 1.11 ± 0.37 | 0.445 |
| eGFR(ml/min/1.73m^2^) | 94.95 ± 20.33 | 85.22 ± 25.61 | <0.001 |
| hs-CRP(mg/L) | 1.70(0.70, 3.40) | 1.30(0.60, 3.50) | 0.309 |
| Albumin(g/L) | 40.89 ± 4.02 | 39.00 ± 4.93 | <0.001 |
| Hb(g/L) | 141.42 ± 16.54 | 133.38 ± 20.08 | <0.001 |
| Lymphocyte(10^9^/L) | 2.06 ± 0.67 | 1.98 ± 0.67 | 0.174 |
| Neutrophil(10^9^/L) | 4.25 ± 1.56 | 4.39 ± 1.62 | 0.320 |
| **Medication** | | | |
| OAD, n (%) | 258 (61.6) | 132 (74.2) | 0.004 |
| Insulin, n (%) | 47 (11.2) | 63 (35.4) | <0.001 |
| ACEI/ARB, n (%) | 73 (18.4) | 42 (24.9) | 0.105 |
| **Malnutrition** | | | |
| GLIM, n (%) | 65 (15.8) | 41 (24.0) | 0.026 |
| PNI, continuous | 51.17 ± 5.56 | 48.88 ± 6.59 | <0.001 |
| PNI, categorical, n (%) | 31 (7.2) | 30 (16.3) | 0.001 |
| NRI, continuous | 102.92 ± 6.73 | 99.91 ± 7.85 | <0.001 |
| NRI, categorical, n (%) |  |  | 0.001 |
| Mild | 50 (11.7) | 27 (14.7) |  |
| Moderate-severe | 77 (18.0) | 56 (30.4) |  |
| CONUT, continuous | 1(0, 2) | 1(0, 2) | 0.007 |
| CONUT, categorical, n (%) |  |  | 0.004 |
| Mild | 99 (23.1) | 58 (31.5) |  |
| Moderate-severe | 13 (3.0) | 13 (7.1) |  |

Abbreviations: DR: diabetic retinopathy; BMI: body mass index; SBP: systolic blood pressure; DBP: diastolic blood pressure; DN: diabetic nephropathy; DPN: diabetic peripheral neuropathy; CAD: coronary artery disease; ASCVD: atherosclerotic cardiovascular disease; FBG: fasting blood glucose; 2hPBG: 2 hours postprandial blood glucose; HbA1c: glycosylated hemoglobin; TG: triglyceride; TC: total cholesterol; LDL-C: low-density lipoprotein cholesterol; HDL-C: high-density lipoprotein cholesterol; eGFR: estimated glomerular filtration rate; hs-CRP: hypersensitive C-reactive protein; OAD:oral antidiabetic drug; ACEI/ ARB: angiotensin-converting enzyme inhibitor/angiotensin receptor blocker; GLIM: global leadership initiative on malnutrition; PNI: prognostic nutritional index; NRI: nutritional risk index; COUNT, controlling nutritional status.

**Supplementary Table 2. Baseline characteristics of malnourished and non-malnourished patients assessed by GLIM.**

|  | Non-malnutrition  （n=477） | Malnutrition  （n=106） | p-value | |
| --- | --- | --- | --- | --- |
| **Demographic characteristics** | | | | |
| Age (years) | 56.09 ± 11.23 | 63.40 ± 12.51 | <0.001 | |
| Age≥60 | 176 (36.9) | 63 (59.4) | <0.001 | |
| Male, n (%) | 283 (59.3) | 79 (74.5) | 0.005 | |
| BMI (kg/m^2^) | 24.56 ± 3.50 | 22.84 ± 2.77 | <0.001 | |
| BMI ≥ 24 kg/m^2^, n (%) | 248 (52.0) | 30 (28.3) | <0.001 | |
| Smoking, n (%) | 132 (27.7) | 31 (29.2) | 0.846 | |
| Low education, n (%) | 397 (83.23) | 88 (83.02) | 0.560 | |
| **Medical history and Clinical condition** | | | |  |
| Hypertension, n (%) | 203 (42.6) | 52 (49.1) | 0.266 | |
| SBP (mmHg) | 132.83 ± 17.85 | 134.08 ± 21.07 | 0.529 | |
| DBP (mmHg) | 82.92 ± 11.12 | 82.01 ± 13.47 | 0.462 | |
| Duration of diabetes (years) | 6.00 (0.50, 10.00) | 8.50 (2.25, 12.75) | 0.017 | |
| DN, n (%) | 58 (12.2) | 24 (22.6) | 0.008 | |
| DPN, n (%) | 206 (43.2) | 45 (42.5) | 0.976 | |
| CAD, n (%) | 32 (6.7) | 11 (10.4) | 0.271 | |
| Stroke, n (%) | 15 (3.1) | 7 (6.6) | 0.159 | |
| ASCVD, n (%) | 45 (9.4) | 16 (15.1) | 0.122 | |
| Anemia, n (%) | 59 (12.4) | 38 (35.8) | <0.001 | |
| **Laboratory examination** | | | |  |
| FBG (mmol/L) | 8.60 ± 3.11 | 8.82 ± 3.47 | 0.524 | |
| 2hPBG (mmol/L) | 11.33 ± 3.92 | 12.07 ± 4.98 | 0.098 | |
| HbA1C (%) | 9.73 ± 2.37 | 10.63 ± 2.50 | 0.001 | |
| TG (mmol/L) | 1.64(1.10, 2.68) | 1.38 (1.00, 2.37) | 0.036 | |
| TC (mmol/L) | 5.24 ± 1.47 | 5.04 ± 1.62 | 0.227 | |
| LDL-C (mmol/L) | 3.25 ± 0.96 | 3.21 ± 1.13 | 0.675 | |
| HDL-C (mmol/L) | 1.11 ± 0.40 | 1.07 ± 0.27 | 0.394 | |
| eGFR (ml/min/1.73m^2^) | 95.24 ± 20.23 | 81.31 ± 25.95 | <0.001 | |
| hs-CRP (mg/L) | 1.30 (0.60, 2.90) | 3.20 (1.10, 9.95) | <0.001 | |
| Albumin (g/L) | 41.28 ± 3.54 | 37.00 ± 5.43 | <0.001 | |
| Hb (g/L) | 141.18 ± 15.97 | 132.31 ± 21.57 | <0.001 | |
| Lymphocyte (10^9^/L) | 2.17 ± 0.63 | 1.58 ± 0.62 | <0.001 | |
| Neutrophil (10^9^/L) | 3.90 ± 1.20 | 5.73 ± 2.05 | <0.001 | |
| **Medication** | | | | |
| OAD, n (%) | 298 (63.8) | 74 (73.3) | 0.090 | |
| Insulin, n (%) | 78 (16.7) | 25 (24.8) | 0.080 | |
| ACEI/ARB, n (%) | 91 (20.7) | 15 (15.3) | 0.285 | |

Abbreviations: DR: diabetic retinopathy; BMI: body mass index; SBP: systolic blood pressure; DBP: diastolic blood pressure; DN: diabetic nephropathy; DPN: diabetic peripheral neuropathy; CAD: coronary artery disease; ASCVD: atherosclerotic cardiovascular disease; FBG: fasting blood glucose; 2hPBG: 2 hours postprandial blood glucose; HbA1c: glycosylated hemoglobin; TG: triglyceride; TC: total cholesterol; LDL-C: low-density lipoprotein cholesterol; HDL-C: high-density lipoprotein cholesterol; eGFR: estimated glomerular filtration rate; hs-CRP: hypersensitive C-reactive protein; OAD:oral antidiabetic drug; ACEI/ ARB: angiotensin-converting enzyme inhibitor/angiotensin receptor blocker; GLIM: global leadership initiative on malnutrition.

**Supplementary Table 3. Baseline characteristics of malnourished and non-malnourished patients assessed by PNI.**

|  | Non-malnutrition  (n=551) | Malnutrition  (n=61) | p-value | |
| --- | --- | --- | --- | --- |
| **Demographic characteristics** | | | | |
| Age (years) | 56.83±11.40 | 64.48±13.02 | <0.001 | |
| Age≥60 | 216 (39.2) | 40 (65.6) | <0.001 | |
| Male, n (%) | 336 (61.0) | 47 (77.0) | 0.014 | |
| BMI (kg/m^2^) | 24.35±3.41 | 23.42±3.75 | 0.046 | |
| BMI ≥ 24 kg/m^2^, n (%) | 271 (49.2) | 23 (37.7) | 0.089 | |
| Smoking, n (%) | 158 (28.7) | 11 (18.0) | 0.078 | |
| Low education, n (%) | 461 (83.7) | 49 (80.3) | 0.507 | |
| **Medical history and Clinical condition** | | | |  |
| Hypertension, n (%) | 239 (43.3) | 34 (55.7) | 0.065 | |
| SBP (mmHg) | 132.43±18.21 | 138.07±21.67 | 0.055 | |
| DBP (mmHg) | 82.76±11.22 | 82.31±15.15 | 0.821 | |
| Duration of diabetes (years) | 6 (1,10) | 7 (2,10) | 0.407 | |
| DN, n (%) | 69 (12.5) | 25 (41.0) | <0.001 | |
| DPN, n (%) | 240 (43.6) | 26 (42.6) | 0.889 | |
| CAD, n (%) | 40 (7.3) | 5 (8.2) | 0.790 | |
| Stroke, n (%) | 18 (3.3) | 5 (8.2) | 0.055 | |
| ASCVD, n (%) | 55 (10.0) | 9 (14.8) | 0.248 | |
| Anemia, n (%) | 78 (14.2) | 33 (54.1) | <0.001 | |
| **Laboratory examination** | | | |  |
| FBG (mmol/L) | 8.62±3.13 | 9.27±3.82 | 0.208 | |
| 2hPBG (mmol/L) | 11.42±4.14 | 12.02±5.02 | 0.375 | |
| HbA1C (%) | 9.80±2.37 | 10.71±2.68 | 0.006 | |
| TG (mmol/L) | 1.10 (0.83,1.61) | 1.41 (0.95,2.42) | 0.129 | |
| TC (mmol/L) | 5.20±1.49 | 4.95±1.62 | 0.212 | |
| LDL-C (mmol/L) | 3.24±0.98 | 3.12±1.13 | 0.365 | |
| HDL-C (mmol/L) | 1.10±0.39 | 1.02±0.27 | 0.119 | |
| eGFR (ml/min/1.73m^2^) | 93.99±20.59 | 74.25±30.08 | <0.001 | |
| hs-CRP (mg/L) | 1.50 (0.60,3.00) | 4.20 (1.90,13.10) | <0.001 | |
| Albumin (g/L) | 41.19±3.46 | 32.48±4.16 | <0.001 | |
| Hb (g/L) | 140.83±16.43 | 122.46±23.07 | <0.001 | |
| Lymphocyte (10^9^/L) | 2.11±0.64 | 1.27±0.39 | <0.001 | |
| Neutrophil (10^9^/L) | 4.21±1.52 | 5.01±1.89 | 0.002 | |
| **Medication** | | | | |
| OAD, n (%) | 351 (63.7) | 41 (62.7) | 0.588 | |
| Insulin, n (%) | 91 (16.5) | 19 (31.1) | 0.005 | |
| ACEI/ARB, n (%) | 102 (20.2) | 13 (22.0) | 0.735 | |

Abbreviations: DR: diabetic retinopathy; BMI: body mass index; SBP: systolic blood pressure; DBP: diastolic blood pressure; DN: diabetic nephropathy; DPN: diabetic peripheral neuropathy; CAD: coronary artery disease; ASCVD: atherosclerotic cardiovascular disease; FBG: fasting blood glucose; 2hPBG: 2 hours postprandial blood glucose; HbA1c: glycosylated hemoglobin; TG: triglyceride; TC: total cholesterol; LDL-C: low-density lipoprotein cholesterol; HDL-C: high-density lipoprotein cholesterol; eGFR: estimated glomerular filtration rate; hs-CRP: hypersensitive C-reactive protein; OAD:oral antidiabetic drug; ACEI/ ARB: angiotensin-converting enzyme inhibitor/angiotensin receptor blocker; PNI: prognostic nutritional index.

**Supplementary Table 4. Baseline characteristics of malnourished and non-malnourished patients assessed by NRI.**

|  | Non-malnutrition  （n=402） | Malnutrition  （n=210） | p-value | |
| --- | --- | --- | --- | --- |
| **Demographic characteristics** | | | | |
| Age (years) | 55.96±11.60 | 60.70±11.55 | <0.001 | |
| Age≥60 | 219(54.5) | 75(35.7） | <0.001 | |
| Male, n (%) | 256(63.7) | 127(60.5) | 0.437 | |
| BMI (kg/m^2^) | 24.86±3.19 | 23.105±3.64 | <0.001 | |
| BMI ≥ 24 kg/m^2^, n (%) | 219(54.5) | 75(35.7) | <0.001 | |
| Smoking, n (%) | 113(28.1) | 56(26.7) | 0.705 | |
| Low education, n (%) | 324(80.6) | 186(88.6) | 0.012 | |
| **Medical history and Clinical condition** | | | |  |
| Hypertension, n (%) | 174(43.3) | 99(47.1) | 0.362 | |
| SBP (mmHg) | 133.49±17.65 | 132.04±20.25 | 0.383 | |
| DBP (mmHg) | 83.70±11.24 | 80.83±12.24 | 0.004 | |
| Duration of diabetes (years) | 6.00(0.67,10.00) | 7.00(1.00,10.00) | 0.372 | |
| DN, n (%) | 42(10.4) | 52(24.8) | <0.001 | |
| DPN, n (%) | 164(40.8) | 102(48.6) | 0.065 | |
| CAD, n (%) | 29(7.2) | 16(7.6) | 0.855 | |
| Stroke, n (%) | 13(3.2) | 10(4.8) | 0.345 | |
| ASCVD, n (%) | 41(10.2) | 23(11.0) | 0.772 | |
| Anemia, n (%) | 34(8.5) | 77(36.7) | <0.001 | |
| **Laboratory examination** | | | |  |
| FBG (mmol/L) | 8.74±3.22 | 8.58±3.18 | 0.555 | |
| 2hPBG (mmol/L) | 11.36±4.00 | 11.70±4.67 | 0.342 | |
| HbA1C (%) | 9.51±2.30 | 11.70±4.68 | <0.001 | |
| TG (mmol/L) | 1.73(1.16,2.77) | 1.37(0.96,2.33) | <0.001 | |
| TC (mmol/L) | 5.29±1.53 | 4.97±1.43 | 0.014 | |
| LDL-C (mmol/L) | 3.29±0.99 | 3.11±1.00 | 0.036 | |
| HDL-C (mmol/L) | 1.10±0.33 | 1.07±0.46 | 0.354 | |
| eGFR (ml/min/1.73m^2^) | 94.78±19.95 | 86.74±25.90 | <0.001 | |
| hs-CRP (mg/L) | 1.45(0.70,3.00) | 1.90(0.70,5.30) | 0.012 | |
| Albumin (g/L) | 42.61±2.74 | 35.95±3.58 | <0.001 | |
| Hb (g/L) | 143.82±15.81 | 129.79±18.51 | <0.001 | |
| Lymphocyte (10^9^/L) | 2.08±0.69 | 1.95±0.69 | 0.023 | |
| Neutrophil (10^9^/L) | 4.17±1.37 | 4.52±1.89 | 0.020 | |
| **Medication** | | | | |
| OAD, n (%) | 263(65.4) | 129(61.4) | 0.328 | |
| Insulin, n (%) | 65(16.2) | 45(21.4) | 0.108 | |
| ACEI/ARB, n (%) | 78(21.0) | 37(19.1) | 0.584 | |

Abbreviations: DR: diabetic retinopathy; BMI: body mass index; SBP: systolic blood pressure; DBP: diastolic blood pressure; DN: diabetic nephropathy; DPN: diabetic peripheral neuropathy; CAD: coronary artery disease; ASCVD: atherosclerotic cardiovascular disease; FBG: fasting blood glucose; 2hPBG: 2 hours postprandial blood glucose; HbA1c: glycosylated hemoglobin; TG: triglyceride; TC: total cholesterol; LDL-C: low-density lipoprotein cholesterol; HDL-C: high-density lipoprotein cholesterol; eGFR: estimated glomerular filtration rate; hs-CRP: hypersensitive C-reactive protein; OAD:oral antidiabetic drug; ACEI/ ARB: angiotensin-converting enzyme inhibitor/angiotensin receptor blocker;NRI: nutritional risk index.

**Supplementary Table 5. Baseline characteristics of malnourished and non-malnourished patients assessed by CONUT.**

|  | Non-malnutrition  （n=429） | Malnutrition  （n=183） | p-value | |
| --- | --- | --- | --- | --- |
| **Demographic characteristics** | | | | |
| Age (years) | 55.13±11.02 | 63.27±11.55 | <0.001 | |
| Age≥60 | 140(32.80) | 115(62.50) | <0.001 | |
| Male, n (%) | 257(60.20) | 125(67.90) | 0.070 | |
| BMI (kg/m^2^) | 24.36±3.46 | 24.01±3.41 | 0.254 | |
| BMI ≥ 24 kg/m^2^, n (%) | 211(49.40) | 83(45.10) | 0.328 | |
| Smoking, n (%) | 129(30.20) | 39(21.20) | 0.022 | |
| Low education, n (%) | 358(83.80) | 151(82.10) | 0.589 | |
| **Medical history and Clinical condition** | | | |  |
| Hypertension, n (%) | 172(40.30) | 101(54.90) | 0.001 | |
| SBP (mmHg) | 132.87±17.73 | 133.29±20.64 | 0.811 | |
| DBP (mmHg) | 83.38±11.21 | 81.18±12.54 | 0.032 | |
| Duration of diabetes (years) | 5(0.17,10) | 10(2,14) | <0.001 | |
| DN, n (%) | 50(11.70) | 44(23.90) | <0.001 | |
| DPN, n (%) | 180(42.20) | 85(46.20) | 0.355 | |
| CAD, n (%) | 21(4.90) | 24(13) | <0.001 | |
| Stroke, n (%) | 8(1.90) | 15(8.20) | <0.001 | |
| ASCVD, n (%) | 29(6.80) | 35(19) | <0.001 | |
| Anemia, n (%) | 49(11.50) | 61(33.20) | <0.001 | |
| **Laboratory examination** | | | |  |
| FBG (mmol/L) | 8.82±3.12 | 8.37±3.40 | 0.107 | |
| 2hPBG (mmol/L) | 11.46±4.09 | 11.52±4.56 | 0.884 | |
| HbA1C (%) | 9.99±2.27 | 9.67±2.72 | 0.137 | |
| TG (mmol/L) | 1.75(1.18,2.75) | 1.25(0.95,2.17) | <0.001 | |
| TC (mmol/L) | 5.58±1.34 | 4.25±1.43 | <0.001 | |
| LDL-C (mmol/L) | 3.49±0.87 | 2.62±1.02 | <0.001 | |
| HDL-C (mmol/L) | 1.13±0.41 | 1.02±0.26 | 0.001 | |
| eGFR (ml/min/1.73m^2^) | 95.71±20.30 | 83.38±24.91 | <0.001 | |
| hs-CRP (mg/L) | 1.50(0.60,3.00) | 1.85(0.70,5.60) | 0.005 | |
| Albumin (g/L) | 41.48±3.26 | 37.65±5.41 | <0.001 | |
| Hb (g/L) | 141.93±15.89 | 132.26±20.78 | <0.001 | |
| Lymphocyte (10^9^/L) | 2.18±0.58 | 1.68±0.74 | <0.001 | |
| Neutrophil (10^9^/L) | 4.13±1.39 | 4.66±1.89 | <0.001 | |
| **Medication** | | | | |
| OAD, n (%) | 261(61.10) | 131(72.20) | 0.017 | |
| Insulin, n (%) | 62(14.50) | 48(26.10) | 0.001 | |
| ACEI/ARB, n (%) | 70(17.80) | 45(26.30) | 0.021 | |

Abbreviations: DR: diabetic retinopathy; BMI: body mass index; SBP: systolic blood pressure; DBP: diastolic blood pressure; DN: diabetic nephropathy; DPN: diabetic peripheral neuropathy; CAD: coronary artery disease; ASCVD: atherosclerotic cardiovascular disease; FBG: fasting blood glucose; 2hPBG: 2 hours postprandial blood glucose; HbA1c: glycosylated hemoglobin; TG: triglyceride; TC: total cholesterol; LDL-C: low-density lipoprotein cholesterol; HDL-C: high-density lipoprotein cholesterol; eGFR: estimated glomerular filtration rate; hs-CRP: hypersensitive C-reactive protein; OAD:oral antidiabetic drug; ACEI/ ARB: angiotensin-converting enzyme inhibitor/angiotensin receptor blocker; COUNT, controlling nutritional status.

**Supplementary Table 6. Univariable logistic regression analysis for**

**variables and DR.**

|  | **OR (95%CI)** | **p-value** |
| --- | --- | --- |
| Age | 1.02(1.00-1.03) | 0.036 |
| Age≥60 | 1.21(0.85-1.72) | 0.281 |
| Male | 0.74(0.52-1.06) | 0.096 |
| BMI | 0.94(0.89-0.99) | 0.023 |
| BMI≥24 kg/m^2^ | 0.79(0.56-1.12) | 0.192 |
| Smoking | 0.59(0.39-0.88) | 0.012 |
| Low Education | 1.24(0.77-2.02) | 0.386 |
| Hypertension | 1.45(1.03-2.06) | 0.035 |
| Duration of diabetes | 1.12(1.09-1.15) | <0.001 |
| DN | 3.62(2.31-5.71) | <0.001 |
| DPN | 3.38(2.37-4.87) | <0.001 |
| CAD | 1.18(0.60-2.21) | 0.620 |
| Stroke | 5.73(2.40-15.13) | <0.001 |
| ASCVD | 2.10(1.24-3.55) | 0.006 |
| Anemia | 2.58(1.69-3.94) | <0.001 |
| FBG | 0.98(0.93-1.03) | 0.448 |
| 2hPBG | 0.99(0.95-1.03) | 0.567 |
| HbA1C | 0.98(0.91-1.05) | 0.588 |
| TG | 0.95(0.86-1.04) | 0.278 |
| TC | 0.93(0.83-1.05) | 0.242 |
| LDL-C | 0.85(0.71-1.01) | 0.065 |
| HDL-C | 1.19(0.75-1.86) | 0.449 |
| eGFR | 0.98(0.97-0.99) | <0.001 |
| hs-CRP | 1.03(1.01-1.04) | 0.005 |
| Albumin | 0.91(0.87-0.94) | <0.001 |
| Hb | 0.98(0.97-0.99) | <0.001 |
| Lymphocyte | 0.83(0.64-1.08) | 0.174 |
| Neutrophil | 1.06(0.95-1.18) | 0.320 |
| OAD | 1.79(1.22-2.66) | 0.003 |
| Insulin | 4.32(2.82-6.69) | <0.001 |
| ACEI/ARB | 1.46(0.95-2.25) | 0.084 |

Abbreviations: DR: diabetic retinopathy; BMI: body mass index; DN: diabetic nephropathy; DPN: diabetic peripheral neuropathy; CAD: coronary artery disease; ASCVD: atherosclerotic cardiovascular disease; FBG: fasting blood glucose; 2hPBG: 2 hours postprandial blood glucose; HbA1c: glycosylated hemoglobin; TG: triglyceride; TC: total cholesterol; LDL-C: low-density lipoprotein cholesterol; HDL-C: high-density lipoprotein cholesterol; eGFR: estimated glomerular filtration rate; hs-CRP: hypersensitive C-reactive protein; OAD:oral antidiabetic drug; ACEI/ ARB: angiotensin-converting enzyme inhibitor/angiotensin receptor blocker.
